# Indonesian Islamic institutions between the foundation and endowment laws: a critical legal analysis

**DOI:** 10.1186/s40064-016-2772-6

**Published:** 2016-07-29

**Authors:** Ibrahim Siregar

**Affiliations:** Institut Agama Islam Negeri Padangsidimpuan (State Institute of Islamic Studies Padangsidimpuan), Jl. H. T. Rizal Nurdin Km. 4,5 Sihitang, Kecamatan Padangsidimpuan Tenggara, Padangsidimpuan, Sumatera Utara Indonesia

**Keywords:** Indonesian law, Islamic law, Islamic institution, Endowment (*waqf*), Foundation (*yayasan*), Conflict, Critical legal analysis

## Abstract

**Background:**

This research-based paper examined the Indonesian foundation and endowment laws in relation to educational and religious institutions which are managed under foundation legal structure. The institutions examined were: 1) The *Pondok Pesantren al-Ansor* Foundation in Padangsidimpuan City; 2) The *Pesantren Dar al-Ma’arif* Education Foundation in South Labuhanbatu Regency; and 3) ​The *Masjid Agung* Foundation in Medan City.

**Methods:**

Using legal sociology and critical legal analysis, data were collected through field research, document study, and in-depth interviews. The documents studied were laws, books, papers, and other related writings relevant to this research. Interviews were conducted with informants obtained from snowball sampling and key person methods.

**Results:**

It was found that in terms of foundation wealth, these institutions can be categorized into three: (1) foundation with founder’s wealth; (2) foundation with endowment wealth; and (3) foundation with both founder’s and endowment wealth. Even though both foundation and endowment legal structures have the same aim of societal welfare, it was found that when they are merged into one legal structure, the foundation becomes more dominant, and there is a risk that the endowment asset’s status become unclear. The asset may be sold or its status may be changed from endowment wealth to foundation wealth. This unclear status may also be caused by conflict of interests among the foundation members and people involved in the foundations. Even when the foundation status is clear, most if not all foundation members violate the rules stipulated by the Foundation Law. The lack of status clarity has caused these institutions to become objects of conflict.

**Conclusions:**

There is a need to position the status of these institutions accurately and it is recommended that the endowment legal structure is used for institutions with endowment wealth.

## Background

Many social and religious institutions in Indonesia were founded by individuals and organizations to realize social goals, such as building educational, health, and religious facilities. In most cases, the founders of these facilities are wealthy enough such that they can set aside a portion of their wealth to build these facilities without expecting any return; in other words, they are of sincere hearts. More often than not, these facilities are built using legal structures in the form of a foundation (*yayasan*), endowment (*wakaf*) in the form an endowment management body (*badan kenazhiran wakaf—BKW*), or a foundation which has endowment assets (*harta wakaf*). If the legal structure is in the form of an endowment management body, Law No. 41 Year 2004 on Endowment (Endowment Law 2004)^1^ must be adhered to in any endowment transactions or endowment asset management. For example, the endowing procedure must be clear, such as when the endower surrenders part of his or her wealth as endowment (endowment pledge—*ikrar wakaf*) and the appointment of endowment manager (*nazhir wakaf*) to maintain and develop endowment wealth according to the purpose of the endower and the endowment (*ghardh al wāqif dan waqf*).^2^ If the legal structure is in the form of foundation (*yayasan*), then the Law No. 16 Year 2001 on Foundation (Foundation Law 2001),^3^ and its subsequent modification, and the Law No. 28 Year 2004 on Foundation (Foundation Law 2004)^4^ must be obeyed. The foundation founders must separate their personal wealth from the foundation wealth, appoint a management team, and set the purpose of the foundation to fulfill social, religious, or environmental goals,^5^ without expecting any return from whatever profit that the foundation may accrue.^6^

Both of the above laws clearly regulate two distinct legal structures: foundation and endowment.^7^ Even though the former legal structure, foundation, originates from the Western tradition, it has in essence the same social welfare purpose as endowment, which originates from the Islamic tradition.^8^ In the Foundation Law 2001 and 2004, it is stipulated that the management of the foundation is composed of the Governing (*Pembina*), the Supervisory (*Pengurus*), and the Executive (*Pengawas*) Boards. It must be noted that under this Law, the founder of the foundation who also acts as members of the Governing Board must not receive any salary directly or indirectly from the foundation. It is also similarly stipulated that any members of the Executive and Supervisory Board related to or affiliated with the founders also cannot receive a salary. As such, it is clear that an individual who intends to build a foundation ideally must have sufficient wealth which can be allocated for the foundation for the purpose of social welfare without expecting any return from the foundation’s wealth.

The concept of endowment in Islam is that of a continuous charity (*‘amal jariah*), in which the endower will be rewarded perpetually for his or her endowment which benefits society at large.^9^ In the Endowment Law 2004, it is clearly stipulated that an endower has surrendered his or her rights to the wealth being endowed for social or religious purpose.^10^ This endowment wealth or asset is managed and developed by an endowment manager. The management can be composed of an individual, a legal structure such as foundation (*yayasan*), or organization such as mass-organization (*organisasi kemasyarakatan*—*ormas*). The endowment manager is responsible to maintain the endowment wealth by administering, managing, and developing the wealth in accordance to the purpose of the endower and the endowment. The Endowment Law 2004 in Article 12 has stipulated that the manager has the right to take not more than 10 % of the endowment management returns to cover the operational cost of the endowment management.

In Article 10 of the same Law, it has been stipulated that if the endowment management is in the form of foundation, it must fulfill the following requirement: the Executive Board of the foundation must be composed of members well-versed in law and endowment; that is a member must be Muslim, Indonesian citizen, of legal age, trustworthy, and has the physical and mental ability to manage the endowment. In addition, the Foundation Law has stipulated that a foundation can possess wealth or asset in the form of endowment. It must be noted that the word endowment can be mentioned after the word foundation. In short, the foundation wealth must originate from endowment and the foundation must manage the endowment to enable the word endowment to be mentioned after the word foundation. Further, Article 26 of Foundation Law 2001 states that the foundation’s wealth comes from the endowment wealth, and as such, the Endowment Law 2004 applies in any issue related to the foundation.

As such, the normative implications when the two legal structures (endowment and foundation) are combined into a social, education, or religious institution needs to be understood. For example, the foundation’s Executive Board members who are related or affiliated with the Governing Board members cannot receive a salary. In contrast, the endowment management members can receive a salary not more than 10 % of the endowment management returns. In addition, a foundation can be dissolved; its wealth transferred into other foundation with the same purpose or transferred to the state to be administered according to the foundation’s purpose.^11^ In contrast, the endowment wealth or asset must be used in accordance with the goal of the endower and the endowment; it cannot be modified or transferred by the endowment managers.^12^

If the endowment is managed under a foundation, the endowment asset should be clearly categorized as endowment inventory under foundation management. This categorization will ensure that when the foundation Executive Board dissolve the foundation for any reason, the endowment asset can be transferred in accordance with the goals of the endowment and the endower. As such, every endowment under a foundation management must have an authentic proof (*bukti otentik*). Further, foundations that have wealth in the form of endowment asset should include the word endowment after the word foundation. However, this inclusion is not mandated in the Foundation Law stipulations. Article 3 Clause 3 of the Government Regulation No. 63 Year 2008 on the Implementation of Foundation Law (Government Regulation 2008)^13^ only states that the word endowment *can* (not must) be added when a foundation’s wealth takes the form of endowment.

Normatively, it should be understood that a foundation’s Executive Board members cannot receive a salary directly or indirectly from the foundation unless they are not related or affiliated with the foundation’s founders. Only members with no relation or affiliation can receive a salary. As such, when an endowment is managed under a foundation, the endowment’s 10 % return (which could be spent as salary) is the property of the foundation, not individual foundation members. Only the foundation as a whole, not any of its individual members, can decide the salary the Executive Board members or other relevant persons.

Ideally, the foundation founders are not given salary directly or indirectly from the foundation. However, in reality many foundations’ practices diverge from this ideal, be the legal structures take the form of pure foundation or foundation with endowment. This paper examines the implementation of both legal structures in North Sumatra, focusing on the following questions: What are the types of foundation in North Sumatra?; What are the foundations’ activities?; What is the motivation of an individual to found a foundation?; Are there any foundation with endowment assets?; Is there a conflict between the Foundation and Endowment Laws on a foundation with endowment assets?; Do the founders and the members of a foundation receive a salary from their foundation?

## Research method

A qualitative research, this study examined the management and types of Islamic institutions with endowment assets under the foundation legal structure in North Sumatra. Using legal sociology and critical legal analysis methods, this study investigated the implementation of Foundation and Endowment laws in these institutions through field research, document study, and in-depth interviews.^14^ The documents studied were laws, books, papers, and other related writings relevant to this research. Interviews were conducted with informants obtained from snowball sampling and key person methods.

## The types of institutions under foundation management

Foundation wealth can be categorized into three: First, founder’s wealth, which later can be declared as endowment wealth; Second, endowment wealth from the very beginning; Third, founder’s and endowment wealth. Foundation activities can be divided into three: social, humanitarian, and religious. In terms of activities, the foundations discussed in this paper are humanitarian and religious foundations. The humanitarian foundation conduct Islamic education activities such as *pesantren* (Islamic boarding school) and *madrasah* (Islamic school), while the religious foundation manage a mosque.

1. The *Pondok Pesantren al-Ansor* Foundation (*Yayasan Pondok Pesantren al-Ansor*) in Padangsidimpuan City

The foundation was founded by *Kyai Haji* (KH.) Sahdi Ahmad Lubis in 1994. In the beginning, the foundation borrowed the classrooms of the South Tapanuli Higher Institute of Islamic Studies (*Sekolah Tinggi Agama Islam Tapanuli Selatan*—STAITA) located in Ade Irma Suryani street in Padangsidimpuan city to conduct its *pondok pesantren* classes. In its first year, the *pesantren* only had 5 students. In 1995, a friend of the founder endowed the *pesantren* with a piece of land, which the foundation used to build a simple classroom. When the classroom was built, the foundation head borrowed a classroom of religious primary school (*madrasah ibtidaiyah diniyah*) at the Manunggang village, 600 m away from the current pesantren location. In 1997, the classroom building was completed at Manunggang village, Southeast Padangsidimpuan District (*Kecamatan*).

The foundation structure was as follows: The Governing Board was composed of only the founder, KH. Sahdi Ahmad Lubis. The Executive and Supervisory Board members of the foundation were held by KH. Lubis’ family members, such as his blood relatives and wife. The activities of the foundation were conducted by the *madrasah tsanawiyah* director and head, positions which were also held by the foundation’s founder, KH. Lubis. After the foundation moved to its new location, with its own building and classrooms, it became more successful. Its pesantren, with *salafiyah* as well as *madrasah tsanawiyah* and *aliyah* curriculum, now has about 1000 students. Also, it now has more teachers in addition to its own alumni; graduates from religious and general Indonesian universities, as well as from Middle Eastern universities, now teach at the *pesantren*.

In addition to the rapid increase in student number, the *pesantren* land has also increased as the foundation founder purchases land around the *pesantren*. What was initially a 1 ha endowed land increased to become an 8 ha endowed land, with a number of buildings used as classrooms, foundation and administration office, hall, teacher’s lounge, student dormitory, common kitchen, library, computer laboratory, mosque, sports arena, and teacher’s houses. There are a number of ponds in the *pesantren*, its slightly sloping land contour planted with many trees, creating a cool and beautiful location. The ponds are used as a practice location for a small fishery industry, with the largest one at about 100 × 60 × 7 m used as the main water source.

The funding source of this foundation comes from Student Fees (*Sumbangan Pembinaan Pendidikan*—SPP), as well as student business units of canteen, general kitchen catering, and a small fisheries industry. The foundation head also actively communicates with donors, local government, and the Ministry of Religious affairs to seek funding as grants to build classrooms, student dormitory, administrator office, road paving, and bridge building at the *pesantren*.

In terms of foundation management, there are several issues regarding the legal status of the foundation Executive Board and the foundation wealth from the perspective of Foundation and Endowment laws. First, the foundation has endowment as foundation asset in addition to the land purchased by the founders. Second, the founder holds three overlapping positions: as members of the Governing and Executive Board, as well as the implementer of the foundation activities. Normatively, the founder is legally at fault for holding these overlapping positions, especially as *madrasah* head the founder obtain salary from the foundation. Third, from the perspective of Endowment Law 2004, the endowed land must be protected by the endowment manager to prevent it from being sold, given, or inherited. However, the endowed land is only pledged verbally by the endower to the foundation head with Endowment Pledge Deed (*Akta Ikrar Wakaf—AIW*) issued by the Official in-charge of Endowment Pledge Deed (*Pejabat Pembuat Akta Ikrar Wakaf*). As such, there is a possibility that the endowed land status may become obscure later and even change into non-endowed status.

Through interview with the founder, it was found out that he has not understood the consequence of using the foundation legal structure for his *pesantren*. He has not understood that the foundation wealth belongs to the foundation, not him as a founder, and as such it cannot be transferred to anyone else, including himself or the members of the Executive and Supervisory Board. Also, he has not understood that any person with relation or affiliation to him cannot receive a salary, directly or indirectly, from the foundation. The founder simply stated that he wished to dedicate his knowledge and energy to educate people with religious knowledge according to his expertise, and that he will make a living through his *pesantren*. Along the way, he became interested to obtain funding from the government, and he was told that he needed to create a foundation legal structure to get this funding. It was then that he visited a public notary to request a Notary Deed on *Pondok Pesantren al-Ansor* (*Akta Notaris Yayasan Pondok Pesantren al*-*Ansor*) without understanding the consequence. He also stated that he was not informed by anyone on this consequence.^15^

2. The ​*Pesantren Dar al-Ma’arif* Education Foundation (*Yayasan Pendidikan Pesantren Dar al-Ma’arif*) in South Labuhanbatu Regency

The foundation main activity is providing Islamic education in the form of *pondok pesantren*. It was founded by the late KH. Abdullah Afandi in 1992. He held the position of the Head of the foundation Executive Board as stated in the Notary Deed No. 45 Year 1992, which was issued before Foundation Law 2001 had taken effect. After the founder passed away, in 2005 the foundation deed was changed to suit the Foundation Law 2001 and 2004. The late KH. Abdullah Afandi has 11 children: 6 sons and 5 daughters. The youngest son had passed away in 1998, hence when Abdullah Afandi passed away, he left 10 children. In the Deed, two of his sons were appointed as members of the Governing Board: Abu Khalil and Fakhruddin. Another two were appointed as members of the Executive Board: *Haji* (H.) Ahmad Rifai and Abdul Hakim, as head and secretary respectively. A grandson, Syahrul Anwar was appointed as treasury, and a son-in-law, H. Rajuddin, was appointed as a Supervisory Board member.

On the other hand, the foundation land has pledged as endowed land by the late KH. Abdullah Afandi before the *Pejabat Pembuat Akta Ikrar Wakaf* in Kotapinang District in 1993. Abu Khalil, his son, was appointed as the endowment manager. Now, he also holds the position of the Governing Board member of the foundation.

In addition to 3 ha of land being endowed formally for the *pondok pesantren*, KH. Abdullah Afandi had also endowed an oil palm plantation to fund the operational cost of the pesantren and the building of its infrastructures, albeit verbally or informally. This informal or verbal endowment has remained until now. The infrastructures built at the pesantren were dormitory, classrooms, and teachers’ housing. The funding source for this infrastructure also came from the community endowment and government grants. As the bulk of the foundation’s wealth comes from endowment, it is advisable that the word endowment (*wakaf*) is added after the word foundation (*yayasan*) to become *yayasan wakaf* and the endowment management should take the form of the foundation, as in accordance with Article 5 of the Government Regulation 2008. Thus, it can become clear that the endowment status is the most dominant in this Islamic education institution.

Recently, there is a significant problem facing the *pesantren*. After 10 years of the founder’s death, many of his heirs view the *pesantren* as inheritance which they can manage in accordance with inheritance norm. They do not legally understand the function of positions in the foundation structure and the status of the endowment asset. As such, the position of the Governing Board member as the highest position in the foundation is ignored by the Executive Board members, who act on his own without following the instruction of the Governing and Supervisory Board members. The Executive Board member always perform activities in contradiction with the Foundation Statute (*Anggaran Dasar Yayasan*), such as doubling up as activity implementer in catering management. The financial report and business unit income is also inaccessible, even though in the Foundation Law it has been stated that Executive Board members who have relation or affiliation with the Governing Board members cannot receive a salary from the foundation, be it directly or indirectly.^16^

Furthermore, there is an unlawful activity performed by the Executive Board member concerning the endowment asset. The *pesantren* location which has been pledged formally by the late founder was not maintained properly; its original borders were disregarded. When a concrete wall was built to enclose the *pesantren*, these borders were ignored such that there was only 7 × 100 m of land left. When the Governing Board member asked the Supervisory Board member about this, the latter answered that when the wall was being built, he has mentioned to the Secretary of the Executive Board the original borders of the *pesantren* land. However, the Secretary ignored the fact and replied that the wall was built as such due to a lack of funds.

Another more serious violation of the Endowment Law 2004 can be found in the change of endowed land status by the Executive Board members. One hectare out of the 3 ha of plantation land endowed by the late endower who was also the foundation founder was sold by the foundation secretary to the public. The land was divided into plots and sold without the knowledge of the endowment manager who also sits on the Governing Board. The sale also did not follow the proper endowment asset transfer procedure. When the endowment manager demanded an explanation from the foundation secretary, the latter claimed that the plantation land did not have an endowment deed and was unsafe from the oil palm theft committed most probably from people around the land. The secretary also claimed that the plantation land has been replaced by another 2 ha of plantation land located close to the pesantren. The manager asked the secretary the sale price of the land plots, but the latter did not answer clearly. The manager found out that the secretary sold the land at 20 million rupiah a plot, netting him a total of 800 million rupiah as there are 40 land plots altogether. The purchase price of the new plantation land was only 500 million rupiah. As such, there is a sum of 300 million rupiah unaccounted by the secretary and not reported to the Governing Board member who is also the endowed land manager.^17^

The above sale also did not involve the Indonesian Endowment Agency (*Badan Wakaf Indonesia*—BWI) as stipulated in the Endowment Law 2004 and Government Regulation No. 42 Year 2006 (Government Regulation 2006).^18^ In the Endowment Law 2004, it has been stipulated in Article 67 Clause 1, that the violator could be jailed for 5 years and/or fined 500 million rupiah.^19^ When the secretary heard this information, he defended himself by saying that the sale did not involve any endowed land, but instead his own land, which happened to be located close by. The manager/Governing Board member then asked the secretary about the relationship between the sold land and the 2 ha of the supposedly replaced land. Many other related questions were asked, but the secretary was not willing to answer clearly. He also refused to attend any meetings with the manager/Governing Board member, who also happens to be his older brother.

From the perspective of foundation law, the secretary has violated the stipulation that he should follow the instruction of the Governing Board member, whose position is doubly strong as the endowment manager. He was told that the funding origin to build the *pesantren* infrastructure must be clear in its origin due to the sacred nature of the *pesantren* as religious education institution. To date, the secretary has used funding from money given by the public as charity due to his position as the caretaker spiritual leader (*murshid*) of the *Naqshabandi Tariqa*, a position which he holds after the death of the last *murshid*, his oldest brother, Shaykh Ahmad Rifai. He claimed that his father also did in his tenure as the foundation Executive Board head.^20^ The Governing Board member/endowment manager then clarified that their father was different from them. KH. Abdullah Afandi was the *murshid* of the *Tariqa*, while his children are not. As such, Afandi had the right to manage the *Tariqa*’s wealth as he sees fit, as well as obtaining a salary out of the management duty. He also had the right to use the wealth for the benefit of the *Dar al-Ma’arif Pesantren*. As the foundation secretary and his other siblings are not the *murshid* of the *Tariqa*, any amount of money used to contribute to the *pesantren* infrastructure must be recorded as debt to be paid to the *Tariqa* in a later time.

Another complication can be found in the view of the foundation founder heir’s that the *pesantren* is part of their inheritance. Most probably because of their legal ignorance, the heirs could not distinguish between inheritance, foundation, and endowment matters. When the secretary was asked about the sale of the 6.5 ha oil palm plantation for 500 million rupiah, he answered that the money was part of his inheritance. He mentioned that their other male siblings were also given the same size of land. The manager then asked that he only inherited 6 ha of empty land, without any plantation, which was then purchased by their older brother for 4 million rupiah in 2005. This amount of money is very little compared to what the secretary has obtained with his supposed land inheritance. The secretary answered that because he is the one who remains in the *pesantren* location to fully manage it, he deserves to have more inheritance.^21^ He mentioned that he has developed 10 ha of oil palm plantation to fund the *pesantren* and build its infrastructures. The manager then stated that the *pesantren* has not been managed as well as it should, for example by increasing the learning and teaching quality, streamlining administration, and strengthening the endowment and foundation legal status. The secretary responded that he is responsible only to manage only the *pesantren*’s infrastructure development,^22^ a statement contrary to his early response of being responsible for the full management of the *pesantren*.

As can be seen from the above elaboration, it is very complicated to manage a foundation with endowment assets, especially if the foundation has family members who sit at the Governing, Supervisory, and Executive Boards. The legal ignorance of the members is compounded by the complex foundation, endowment, and inheritance law and norms. In the *Pesantren Dar al-Ma’arif*’s case, conflicts have indeed occurred when the members act not in accordance with the normative stipulations contained in the Foundation and Endowment Laws. If not dealt correctly and sensitively, the conflict may escalate and become intractable, causing damage to the good works which have been done to build the *pesantren*.

3. The​ Masjid Agung Foundation (*Yayasan Masjid Agung*) in Medan City

The *Masjid Agung* of North Sumatra, which was founded in the 1960s, is located on the Diponegoro Street. It has an area of 43 × 43 m, with a tower building and office facility. A large parking space ensures the comfort of this mosque’s visitors, who mostly come with their vehicles. The land for Masjid Agung was endowed by the Bukit Barisan Commander of the Regional Military Command (*Pangdam Bukit Barisan*).^23^ At the time of endowment, the Endowment Law 2004 had not yet been passed, hence the land endowment has not been formalized in the form of Endowment Pledge Deed. Later, some members of the public felt the need to create a foundation to manage the mosque. dr. Fanani Lubis was the Head of the Executive Board and Dr. Abdullah Syah was a member of the same board. The latter resigned in the 1990s and requested the management of the mosque be transferred to the Governor of North Sumatra, as he felt there were improprieties in the foundation which managed the mosque.

However, the Head of the Foundation refused to transfer the mosque management to the Governor. A few years ago, a conflict occurred regarding the mosque management, which later became very public. The public was worried that the unclear status of the mosque’s land can obscure its endowment status, which so far has only been mentioned verbally by the foundation members.^24^ There was also a concern regarding the income and expenditure of the foundation. Some foundation members suspected that the foundation treasury was improperly managed, and hence requested that the foundation return to its mission of serving the public through social and religious services, instead of benefitting only the foundation members. It was reasoned that that as the foundation wealth comes from endowment, the word endowment should be mentioned after the word foundation and the endowment management is handled by the foundation as a whole, in accordance with Article 3 Clause 3 and 4 of the Government Regulation 2008. In addition, it was found that the foundation has for years managed the mosque without the Mosque Management Body (*Badan Kemakmuran Masjid* - BKM).^25^

A later *Bukit BarisanPangdam* thus took an initiative to resolve the conflict. A Resolution Team was formed, which members consisted of the Mayor of Medan, the Indonesian Endowment Agency (*Badan Wakaf Indonesia*—BWI) of North Sumatra, and the Indonesian Ulama Council (*Majelis Ulama Indonesia*—MUI) of Medan City. The BWI head recommended that the former foundation should be dissolved and a management committee in the form of Endowment Management Body should be formed to manage the mosque. This recommendation was agreed by all the Team members. In May 2015, a BKW of the mosque, composed of several distinguished individuals, was formed before a Public Notary.^26^ The Medan Mayor was appointed as the Head of the Executive Board, and members consisted of MUI Medan scholars and other notable individuals.

## Discussion and analysis

From the above elaboration, it can be seen that there are three types of foundation wealth: (1) One with founder’s wealth, which later can be declared as endowment wealth; (2) One with endowment wealth from the very beginning; (3) One with founder’s and endowment wealth. In time, this wealth usually increases through benefactor donation or government grants. In terms of activities, there are two types of foundation covered in this paper, one with humanitarian activities such as in education, and one with religious activities, such as in mosque management. In terms of land, one foundation (*Pondok Pesantren al-Ansor* Foundation) has both founder’s land and endowment land. The founder initially endowed the pesantren with 1 ha of land, which later grew to become 8 ha. Another foundation, the *Pesantren Dar al-Ma'arif* Education Foundation, was initially endowed with 6 ha of land, 3 ha as pesantren location and 3 ha as oil palm plantation to fund the foundation operational activities. The other foundation, the *Masjid Agung* Foundation, has land endowed by the *Pangdam Bukit Barisan*. The mosque infrastructure was later built from benefactors’ donation and local government grant.

In general, the founders mentioned above are not well-versed in both foundation and endowment laws. They do not understand that the founders or anyone related or affiliated with the founders cannot receive a salary directly or indirectly from their foundation. Their motivation to create a foundation was initially to obtain government grants to develop their *pesantrens*. In addition, all the above foundations were founded before the Foundation Law 2001 and 2004 were in effect. As such, practices which may have been legal before these laws were in effect may no longer be so.^27^

It can thus be concluded that there is a gap between a foundation’s ideal and it’s practice. Even the heads of the foundations interviewed in this paper admitted that they did not realize the implication of having their institution under the foundation legal structure. The idealistic foundation legal structure, in which the founder and his or her relatives cannot receive a salary from the foundation, as compared to the more practical endowment legal structure, in which 10 % of the endowment return can be used as salary by the endowment management, should actually make setting up a foundation to be a difficult endeavor. As such, a notary should not accept requests to set up foundation legal structure so easily.^28^ This problem is compounded by the fact that it is well known that many people have used the foundation legal structure for commercial purpose and tax avoidance, without receiving any sanction from the law enforcement.^29^ As such, it needs to be emphasized a foundation should be administered according to the prevailing law. There needs to be a verification process on the worthiness of an individual or a group to set up a foundation, as well as sanctions when they prove to violate the Foundation Law. Only then the law can be said to have performed its functions: social anticipation, social control, and social engineering.^30^

The divergence of ideal and practice in foundations have been a very common occurrence in Indonesia. One solution perhaps can be in the revision of the Foundation Law to allow the founder and his or her family working in the foundation to receive a salary. This paper has shown that some founders’ relatives acted as if their foundation’s wealth is their personal wealth, be it as part of inheritance or because of their management duty. There have also been many conflicts in Indonesia regarding the distribution of many foundations’ wealth, which in many cases are against the foundation laws.^31^

The legal revision of Foundation Law is more appropriate than letting the gap between its ideal and practice to continue. In this context, it can be seen that the use of endowment management is more appropriate as the management can work and manage the endowment professionally with adequate salary. An institution which has endowment wealth should use Endowment Management Body legal structure instead of the foundation legal structure. So, the recommendation of the North Sumatra BWI to change the *Masjid Agung* management from foundation to BKW should be followed by other related Islamic institutions discussed in this paper.

## Conclusion

The Islamic tradition of endowment has long been practiced in the archipelago and has even been institutionalized in the Endowment Law 2004 and Government Regulation 2006. The Western tradition of foundation has also been institutionalized in Indonesia in the Civil Code (KUHPer—*Kitab Undang*-*undang Hukum Perdata*),^32^ and Foundation Law 2001 and 2004. Based on the findings of this research, it is recommended that religious, social, or humanitarian institutions founded by Muslims should be managed under the Endowment Management Body legal structure, instead of the foundation legal structure. The foundation legal structure is often misused to avoid tax and enrich its founders, among other violations. The prohibition of salary to members of the Governing, Executive, and Supervisory Board who have worked hard to manage the foundation create many ‘moral hazards’ problems. As elaborated above, it can be seen that Islamic institution founders and religious figures, such as ulama or pesantren heads, who use the foundation legal structure may always violate the foundation law as long as they continue to make a living from the foundation. Viewed from this perspective, the legal structure of endowment is more rational, as the endowment managers can receive a professional salary from the endowment returns. No matter the type of endowment, general or specific, for eternity or with time-limit,^33^ provision of professional salary has been accommodated legally in Laws and Government Regulations of Indonesia.^34^

## Abbreviations

AIW: *Akta Ikrar Wakaf* (Endowment Pledge Deed)
APBN: *Anggaran Pembangunan dan Biaya Negara* (State Budget)
BKM: *Badan Kemakmuran Masjid* (Mosque Management Body)
BKW: *Badan Kenazhiran Wakaf* (Endowment Management Body)
BPK: *Badan Pemeriksa Keuangan* (Financial Audit Agency)
BWI: *Badan Wakaf Indonesia* (Indonesian Endowment Agency)
KH: *Kyai Haji*
Kodam: *Komando Daerah Militer* (Regional Military Command)
KUHPer: *Kitab Undang*-*undang Hukum Perdata* (Civil Code*)*
MUI: *Majelis Ulama Indonesia* (Indonesian Ulama Council)
Ormas: *Organisasi kemasyarakatan* (mass-organization)
Pangdam: *Panglima Kodam* (Commander of the Regional Military Command)
SPP: *Sumbangan Pembinaan Pendidikan* (Student Fees)
STAITA: *Sekolah Tinggi Agama Islam Tapanuli Selatan* (South Tapanuli Higher Institute of Islamic Studies)

## Footnotes

1 In Indonesian, the law is titled *Undang*-*undang No. 41 Tahun*^2004^*tentang Wakaf*.

2 See Endowment Law 2004, Chapter 1, Article 1, Clause 2: An endower (*wakif*) is a person who surrenders his or her wealth as endowment (*wakaf*); Clause 3: Endowment Pledge (*Ikrar Wakaf*) is an endower’s statement in oral and/or written form to the endowment manager (*nazhir wakaf*) stating the surrender of his or her wealth as endowment; Clause 4: *Nazhir* is a person who accepts endowment wealth (*harta benda*) from the endower to be managed and developed accordingly.

3 In Indonesian, the law is titled *Undang-undang No. 16 Tahun*^2001^*tentang Yayasan*.

4 In Indonesian, the law is titled *Undang-undang No. 28 Tahun*^2004^*tentang Yayasan.*

5 See Foundation Law 2001, Chapter 1, Article 1, Clause 1: A foundation is a legal body which manage wealth independently to achieve certain purposes in social, religious and humanitarian fields.

6 See Foundation Law 2004, Chapter 1, Article 5, Clause 1: A foundation’s wealth, be it in money, commodity, or other forms, is prohibited to be transferred or distributed directly or indirectly, be it in salary, fee, stipend, or other forms with monetary values, to the Governing, Executive, and Supervisory Board Members.

7 This phenomenon has been succinctly summed up by legal experts as ‘legal pluralism’. See John R. Bowen, *Islam, Law, and Equality in Indonesia*, Cambridge: Cambridge University Press, 2003, pp 3–4.

8 For more on legal pluralism, see Lawrence M. Friedman, *Law and Society: An Introduction,* New Jersey: Prentice Hall, 1977, p. 71.

9 See Michael Dumper, *Wakaf Muslim di negara Yahudi*, Jakarta: Lentera, 1999, p. 2. This book is a translation of Professor Dumper’s original English book, *Islam and Israel: Muslim religious endowments and the Jewish state,* Washington DC: Institute for Palestine Studies, 1994.

10 See Endowment Law 2004, Chapter 1, Article 1, Clause 1: *Wakaf* is the legal action of a *wakif* to separate and/or surrender parts of his or her wealth to be used indefinitely or in a duration of time according to the purpose of worship and/or welfare according to the *sharia* (Islamic law).

11 See Foundation Law 2004, Article 68.

12 See Endowment Law 2004, Article 44.

13 In Indonesian, the regulation is titled *Peraturan Pemerintah Republik Indonesia Nomor 63 Tahun*^2008^*tentang Pelaksanaan Undang-Undang Tentang Yayasan*.

14 William M. Evan, *The Sociology of Law*, New York: Free Press, 1980, pp. 12–13.

15 Interview with KH. Sahdi Ahmad Lubis on 20 June 2015.

16 Article 5 of Foundation Law 2004.

17 Interview with Syahrul Anwar, Treasurer of the foundation in April 2015.

18 In Indonesian, the regulation is titled *Peraturan Pemerintah Nomor 42 Tahun*^2006^*tentang Pelaksanaan Undang-Undang Nomor 41 Tahun*^2004^*Tentang Wakaf.*

19 In *fiqh terms*, this land sale is also against most ulama’s *fatwa*. See Muhammad Abid Abdullah Al-Kabisi, *Hukum Wakaf: Kajian Kontemporer Pertama dan Terlengkap tentang Fungsi dan Pengelolaan Wakaf serta Penyelesaian atas Sengketa Wakaf* (tr.), Jakarta: IIMaN, 2003, pp. 349–350. This book is a translation of al-Kabisi’s original Arabic book, *Ahkam al-Waqf fi Al-Syari’ah Al-Islamiyah*.

20 Interview with Abdul Hakim, Secretary of the foundation in May 2013.

21 Information from Syahrul Anwar.

22 Interview with Mahmudin, sibling of the Secretary, May 2015.

23 Information from Prof. Dr. H. Hasan Bakti Nasution, MA., Secretary of the MUI North Sumatra. *Kodam* in Indonesian is an abbreviation for *Komando Daerah Militer* (Regional Military Command), while *panglima* means army commander. Hence, *Panglima Kodam* or *Pangdam* means Commander of the Regional Military Command. *Bukit Barisan* is one of the 14 military command regions of the Indonesian army, named after the hilly mountainous area of North Sumatra.

24 Interview with Hasan Maksum, Secretary of MUI Medan, 21 June 2015.

25 See, Editorial, “*BKM Kembalikan Masjid Agung untuk Umat*” in Waspada Daily, Sunday, 16 August 2015, p. 1 and A2, col. 1.

26 Information from Prof. Dr. H. Muhammad Yasir Nasution, Head of *Badan Wakaf Indonesia* (BWI) North Sumatra, April 2015.

27 The root of the foundation legal structure is dubious. Soeharto set up the Super Semar foundation by force. It has only two members, including him. Soeharto started by gathering 18 university rectors and demanding that they provide 100 names as scholarship recipients. This foundation, which was founded in 1974, was recently fined more than 4 trillion rupiah, payable to the state, because of its loss in court. The Office of the Attorney General (*Kejaksaan Agung*) sued the Foundation as it misuse scholarship money from state banks by transferring it to companies owned by Suharto’s cronies. The Supreme Court (*Mahkamah Agung*) obligated the foundation to pay the fine in August 2015. See, “*Akal Super Semar*” in Tempo Magazine, 24–30 August 2015 edition, p. 12. See also, “*Beasiswa Amal Super Semar*”, in Tempo Magazine, 17 August 1974 edition.

28 Not a few notaries recommend their clients to use the foundation instead of the association legal structure. The former is deemed to be more legally enforceable, even though the latter has actually been regulated formally, as can be found in “stb 1870-64 on Legal Association” [in Indonesian, *Staatsblad*^1870^*No. 64 tentang Perkumpulan-perkumpulan Berbadan Hukum (Rechtspersoonlijkheid van Vereenigingen)*]. Compare with Ibrahim Assegaf and Eryanto Nugroho (2003: 37).

29 See Danang Widoyoko (2003: 22–23), “*UU Yayasan: Legalisasi Bisnis Militer*?” in Jentera: Jurnal Hukum, Jakarta, Pusat Studi Hukum & Kebijakan Indonesia, 2nd Edition.

30 See Ali Zainuddin, *Sosiologi Hukum*, Jakarta: Sinar Grafika, 2006. See also Nur Ahmad Fadhil Lubis, *A History of Islamic Law in Indonesia*, Medan: IAIN Press, 2000, p. 147.

31 KH. Sa’di Ahmad, a founder of Islamic education foundation stated that there have been no precedent of legal cases problematizing the foundation Governing, Executive, and Supervisory members from receiving a salary as compensation for the foundation management. To understand this issue comprehensively, the politics of the Foundation Law needs to be understood. First, there have been many societal and legal demands to prosecute foundation violations in the form of misuse of foundation legal structure for commercial purposes or money laundering. Some prominent cases included foundations founded by Soeharto, as well as foundations founded by the military and other state institutions as a tool for non-budgetary fund-raising. Second, the statement of the Government of Indonesia in the International Monetary Fund’s Letter of Intent to fulfill the latter’s loan requirement. The government has admitted that much state funding came in the form of foundation activities. As the funds were outside the State Budget (*Anggaran Pembangunan dan Biaya Negara*—APBN), in what was usually called non-budgetary fund, their origin and use escaped the scrutiny of Financial Audit Agency (*Badan Pemeriksa Keuangan*—BPK). This is ironic because the intent of the Foundation Law creation was initially to allow BPK to examine and audit foundations. Instead, when it was passed, the Law only demanded foundations to be audited by Public Accountant (Article 52 Clause 3 of Foundation Law 2001). As such, the Law negated BPK’s authority to audit foundations in Indonesia. See Ibrahim Assegaf and Eryanto Nugroho (2003: 36).

32 Before the issuance of Law No. 16 Year 2001, foundation was regulated by Articles 365, 900, 1680 of the Civil Code (KUHPerdata, pasal 365, 900, 1680), which originated from the Netherlands.

33 In Article 1 (1) of the Endowment Law 2004, it is stated that an endowment is a legal act of the endower to separate and/or surrender parts of his or her wealth to be used in perpetuity or for a certain amount of time in accordance with its purpose of religious worship and/or public welfare according to the sharia. See Jaih Mubarok, *Wakaf Produktif*, Bandung: Simbiosa Rekatama Media, 2008a, b, pp. 74–75.

34 Government Regulation Number 42 Year 2006 in Articles 30 (4), (5), and (6) stipulated that the endower’s will can be in the form of *khairi* (general) or specific (*ahli*) endowment. The latter is meant for the welfare of the endower’s blood relatives (*nasab*). In the case that all the relatives has passed away, the endowment status changes to *khairi* which purpose is set by the relevant minister based on the recommendation of the Indonesia Endowment Agency (*Badan Wakaf Indonesia*—BWI).
